# Identification of miRNAs associated with dark-induced senescence in *Arabidopsis*

**DOI:** 10.1186/s12870-015-0656-5

**Published:** 2015-11-04

**Authors:** Xiaoying Huo, Chao Wang, Yibo Teng, Xunyan Liu

**Affiliations:** College of Life and Environmental Sciences, Hangzhou Normal University, Hangzhou, 310036 P. R. China

**Keywords:** *Arabidopsis thaliana*, Dark-induced senescence, Microarray, miRNA

## Abstract

**Background:**

microRNAs (miRNAs) are endogenous small (~21 nucleotide) single-stranded non-coding RNAs that typically function by guiding cleavage of target genes. To find the miRNAs that may be involved in dark-induced leaf senescence, we identified miRNAs by microarray platform using *Arabidopsis thaliana* leaves from both whole darkened plants (DPs) and individually darkened leaves (IDLs).

**Results:**

We found that the expressions of 137 miRNAs (*P* < 0.01, signal intensity >0) were significantly changed both in DP and IDL leaves. Among them, the expression levels of 44 miRNAs were relative higher than others (*P* < 0.01, signal intensity >500). Of these differentially expressed miRNAs, 6 miRNAs (miR319a, 319c, miR159, miR164a, miR164c and miR390a) have been previously reported to be involved in dark-induced leaf senescence, and the remaining 38 miRNAs have not been implicated in leaf senescence before. Target genes of all 44 miRNAs were predicted, and some of them, such as *NAC1*, *At3g28690*, *At2g17640* and *At2g45160*, were found in the Leaf Senescence Database (LSD). GO and KEGG analysis of 137 miRNAs showed that the predicted target genes were significantly enriched in transcription regulation, development-related biological processes and metabolic pathways. Expression levels of some of the corresponding miRNA targets (*At1g73440*, *At2g03220 and At5g54810*) were analysed and found to be significantly different in DP/IDL than that in WT.

**Conclusions:**

A microarray analysis about dark-induced miRNAs involved in leaf senescence are present here. Further expression analysis revealed that some new founding miRNAs maybe regulate leaf senescence in *Arabidopsis*, and the findings highlight the important role of miRNAs in dark-induced leaf senescence.

**Electronic supplementary material:**

The online version of this article (doi:10.1186/s12870-015-0656-5) contains supplementary material, which is available to authorized users.

## Background

Senescence in plants is an intrinsic, genetically determined, natural developmental programme that operates at the end of leaf, fruit, or flower development [1]. It is characterized by the visible yellowing (Chlorophyll degradation) of leaves accompanied by the mobilization of leaf nutrients to the reproductive structures, and is a complex process involving changes of physiological, biochemical and gene expression regulated by endogenous and exogenous factors [2]. As senescence occurs at the ultimate stage in leaf development and precedes cell death [2], it has a crucial impact on agriculture, especially in crops where crop yield is enhanced by longer growth periods. Although leaf senescence is controlled mainly by developmental age, it can be modulated or triggered by adverse environmental factors such as temperature, high salinity, drought, submergence, ozone, constant darkness, nutrient deficiency, light and pathogen infection [2–8]. Therefore, an understanding of leaf senescence mechanisms is important not only for answering fundamental scientific questions but also for increasing crop yields by prolonging photosynthetic activity and minimizing post-harvest quality loss in vegetables [9].

Senescence-like phenomena can also be induced by incubation in darkness [5]. In some ways, dark-induced senescence programs share many common pathways with natural age-dependent senescence [10]. There are some similar symptoms and molecular components in the two conditions, with the exception of ROS production in mitochondria which increases markedly in dark-induced senescent pea leaves [11] and aged potato (*Solanum tuberosum*) tubers [12].

Comparative transcriptome analysis revealed that natural age-dependent and dark-induced senescence regulates overlapping but different sets of genes in *Arabidopsis* rosette leaves [10, 13]. The number of specific genes induced by natural age-dependence is much higher than the number of specific genes induced by darkness [10]. However, transcriptome data of plant leaves undergoing different types of senescence indicated significant differences in gene expression profiles and signalling pathways under the two conditions (natural or darkness) [13]. Furthermore, Keech *et al.* found that the regulation of metabolism differed significantly between an individually darkened leaf (IDL) attached to a whole plant and an equivalent leaf from an entirely darkened plant (DP), though leaf senescence was induced by darkness in both cases [14].

miRNAs are endogenous small (~21 nucleoutide) single stranded non-coding RNAs, which are capable of regulating gene expression via post transcriptional or post translational mechanisms present in nearly all eukaryotes [15–17]. Numerous studies have demonstrated that miRNAs are implicated in most of the essential biological processes in plants, including regulation of development, cell proliferation, apoptosis, signal transduction, hormone and stress responses [18–22]. Recently, some evidence has indicated that miRNAs are effective in regulating different mechanisms entailing plant senescence [6, 23–25].

In recent years, tremendous advance has been made in understanding how senescence functions in plants. Plant senescence is a fascinating and challenging research attracting scientists to investigate this multifaceted phenomenon from different angles [1]. Combined with genetic approaches, senescence in leaves has been studied in the model plant *Arabidopsis thaliana*, with research mainly focusing on senescence-associated gene (SAG) expression and function [13, 26]. To date, several studies have explored the potential involvement of miRNAs in plant senescence. For example, miR319 positively controls leaf senescence by regulating the activity of TCP transcription factors [24]. Overexpression of miR164 represses EIN3-induced early-senescence phenotypes in *Arabidopsis thaliana* leaves [23, 25]. However, knowledge of the role of miRNAs in response to leaf senescence is still limited, with only a few miRNAs characterized for their *in vivo* functions during this process [6, 23–25].

In this study, we aimed to identify miRNAs playing a role in dark-induced leaf senescence of Arabidopsis by using miRNAs microarray platform on DPs and IDLs. Of those identified, eight were further validated experimentally by quantitative real-time PCR (qPCR) and the results were found to be consistent approximately with microarray. The fine-scale expression analysis of miRNA targets responsive to dark-induced senescence provided molecular evidence for the potential involvement of certain miRNAs in dark-induced senescence. Together, the identification of miRNAs and their targets responsive to dark-induced senescence could help to uncover the molecular mechanisms of dark-induced leaf senescence.

## Methods

### Plant material and growth conditions

*Arabidopsis thaliana* (Heyn.) ecotype Columbia (Col-0) seeds were surface sterilized and cold-treated for 3 d at 4 °C. They were then planted in soil and grown ~4 weeks in a controlled environment growth chamber with a long-day photoperiod (16 h light/8 h dark), irradiance of 250 mmol m^−2^ s^−1^, relative humidity of 55 % and a day/night temperature of 22 °C.

### Dark induction of senescence (IDLs and DPs treatment)

**Fig. 1:**
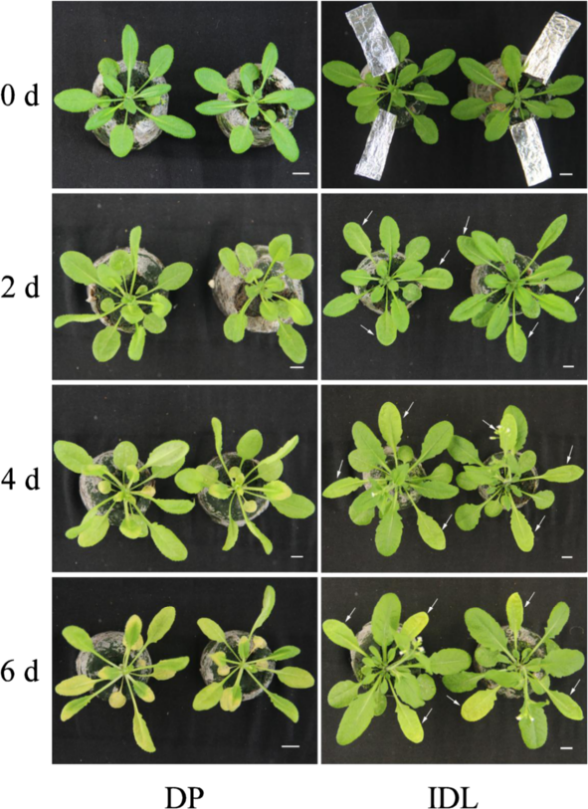
Experimental system and dark-induced senescence phenotype of leaves. Leaves from the 6th to 12th rosettes of plants were induced to senesce by dark-treatment for 2, 4 or 6 d. 0 d before treatment represents WT. For DP, the entire plant was maintained in constant dark. For IDL, the leaves on the rest of the plant remained in the light. White arrows indicate the covered leaves

Leaf senescence was induced in *Arabidopsis* according to the experimental design of Keech et al. [14] (Fig. 1). Leaves in 28 plants from the 6th to 10th rosettes were covered by a black plastic bag and aluminum foil to reduce heat, whereas the rest of the plant remained in light, i.e. IDLs attached to whole plants*.* Leaves were darkened for 2, 4 or 6 d.

In every biological repeats, 28 plants in pots were covered by a black plastic box ventilated from below to allow gas exchange, but still keeping the plants fully darkened, i.e. DPs. Plants were darkened for 2, 4 or 6 d in the same climate chambers as the IDLs treatment. And leaves from the 6th to 10th rosettes without any dark treatment (16 h light/8 h dark, 250 mmol m^−2^ s^−1^) were collected as a control (WT, i.e. DP/IDL-0 d in Fig. 1).

### Chlorophyll (Chl) content determination

The procedure was carried out at 4 °C and dark. A leaf sample (0.25 g) was mashed in a mortar and pestle with 5 ml 80 % acetone (v/v), the extract was filtered through two layers of nylon and added to 25 ml with 80 % acetone. Then it was centrifuged in sealed tubes at 15,000 × g for 5 min. The supernatant was collected and read at 663 and 645 nm for Chl a and Chl b, respectively. The concentrations for Chl were calculated according to the equations of Arnon [27].

### Total RNA and small RNA isolation

Total RNA was extracted using the Trizol reagent (Invitrogen, USA) according to the manufacturer’s protocol. Total RNA quantity and purity were assayed with the NanoDrop ND-1000 spectrophotometer (NanoDrop, USA) at 260/280 nm (ratio > 2.0). Small RNA fractions of 10–40 nucleotides were isolated from the total RNA pool with a Novex 15 % TBE-Urea gel (Invitrogen, USA).

### μParaflo™ MiRNA microarray assay

Microarray assay was performed using a service provider (LC Sciences). Firstly, 4–8 μg total RNA sample were 3’-extended with a poly(A) tail using poly(A) polymerase. An oligonucleotide tag was then ligated to the poly(A) tail for later fluorescent dye staining. Hybridization was performed overnight on a μParaflo microfluidic chip using a micro-circulation pump (A tactic Technologies) [28, 29]. On the microfluidic chip, each detection probe consisted of a chemically modified nucleotide coding segment complementary to target miRNA (from miRBase, http://www.mirbase.org/) or other RNA (control or customer defined sequences) and a spacer segment of polyethylene glycol to extend the coding segment away from the substrate. The detection probes were made by *in situ* synthesis using photo generated reagent (PGR) chemistry. The hybridization melting temperature was balanced by chemical modifications of the detection probes. After RNA hybridization, tag-conjugating Cy3 dye was circulated through the microfluidic chip for dye staining. Fluorescence images were collected using a GenePix 4000B laser scanner (Molecular Device, USA) and digitized using Array-Pro image analysis software (Media Cybernetics). Data were analysed by normalizing the signals using a LOWESS filter after subtracting the background (locally weighted regression) [30].

### Real-time quantitative PCR

The expression of eight selected miRNAs was assayed in DP, IDL and wild-type *Arabidopsis thaliana* (Col-0) by Platinum SYBR Green based qPCR (Invitrogen, 11733–038) with the High-Specificity miRNA QuantiMir RT Kit (RA610A-1, System Biosciences) on ABI 7900. The primers of eight selected miRNAs and internal control gene (UBQ6-1) are available in Additional file 2: Table S1.

The expression of 12 selected genes, such as WRKY22, WRKY53, SAG12, SAG20, was assayed in seven samples by SYBR®Green Real time PCR (TOYOBO, Japan) with the SYBR®Green Realtime PCR MasterMix kit (TOYOBO, Japan) on Eppendorf realplex^4^. The primers of 10 genes and one internal control gene (ACT2, AT3G18780) are available in Additional file 2: Table S1.

### Gene ontology (GO) and pathway analysis

We performed GO analysis on target genes of miRNAs (*P* < 0.01) with differential expression based on the GO database (http://www.geneontology.org/). Pathway analysis was also performed on target genes of the differentially expressed miRNAs based on the KEGG database (http://www.genome.jp/kegg/). Using a *P*-value of <0.5, we determined the enriched pathways.

## Results

### Phenotype and Chl content analysis in the DPs and IDLs in *Arabidopsis*

Upon dark treatment for 2 d, no increase in yellowing was observed in either DPs or IDLs, although leaves began to lose pigment. After dark treatment for 4 d, some older leaves of the DPs showed increased yellowing, whereas IDLs were still pale green with no visible yellowing. At day 6, almost all leaves were yellowing, long and thin in DPs, whereas only some of the treated IDLs showed yellowing (Fig. 1).

**Fig. 2:**
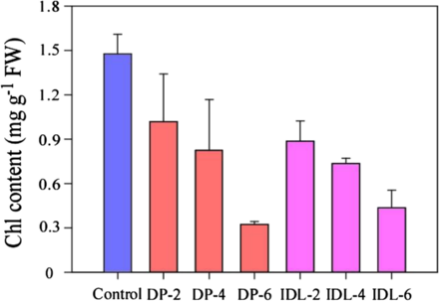
Chlorophyll content change in the control, DP and IDL of *Arabidopsis* leaves. Chl content was detected in leaves with DP/IDL treatment for 0, 2, 4 or 6 d. Error bars indicate SD obtained from four biological repeats

Figure 2 showed that DP/IDL leaves have different Chl content compared with Control (Fig. 2). It was 1.48 mg g^−1^ FW of Chl in Control before treatment. After dark treatment for 2 d, Chl content of DP and IDL leaves is 1.02 and 0.89 mg g^−1^ FW, respectively. At 4 d, DP and IDL leaves decreased to 0.82 and 0.74 mg g^−1^ FW. Moreover, DP and IDL leaves contained around 0.32 and 0.44 mg g^−1^ FW, respectively, after 6 d corresponding treatment.

### Microarray analysis of miRNAs expression in the DPs and IDLs in *Arabidopsis*

**Fig. 3:**
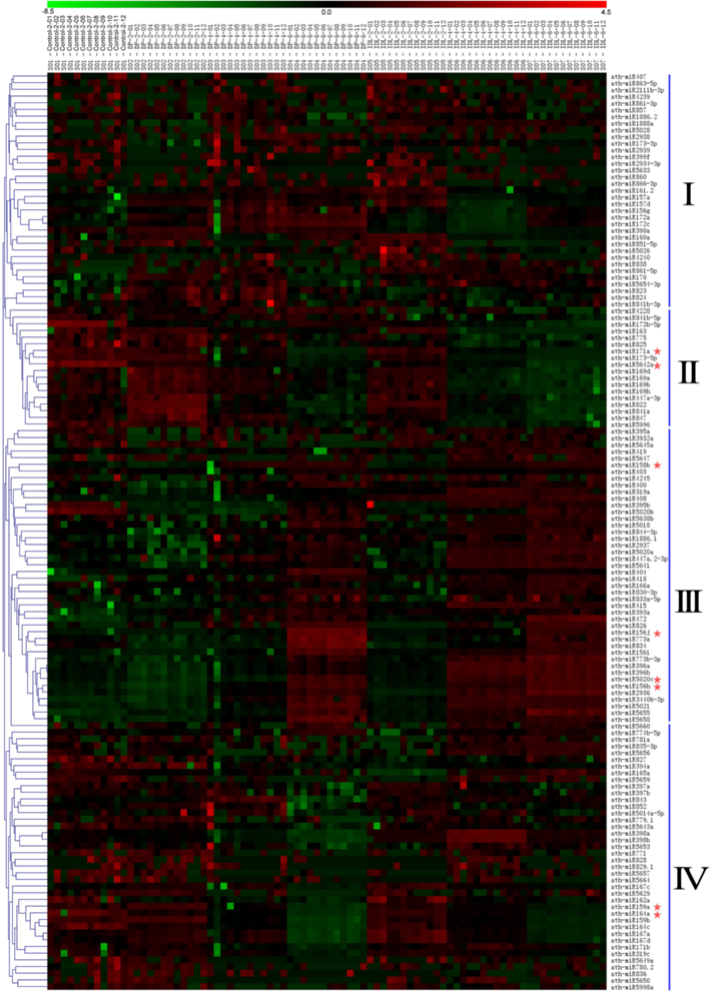
Comparison of the expression patterns of miRNAs in the control, DP and IDL of *Arabidopsis* leaves. Microarray at the *P* < 0.01 level were cluster analysed. The color scale is based on the *Z*-value of the log2 detected signal of miRNAs in samples, from green (relatively low expression) to red (relatively high expression). The heat maps presented here summarize four distinct expression patterns over the time course after dark-induced treatment or IDL treatment

To examine differential expression of miRNAs in *Arabidopsis* between the pre-treated and treated leaves (DPs and IDLs), miRNA microarray analysis was performed to detect the global expression of miRNAs in the DPs and IDLs. Transcript data were statistically significant but had low signals (*P* < 0.01, signal intensity > 0), based on the *Z*-values of the log2 data (data not shown), which were averaged from the two color reversal hybridization experiments (Fig. 3). We found that expression levels of 44 miRNAs significantly changed in DPs and IDLs (*P* < 0.01, signal intensity >500, Additional file 2: Table S2). The miRNA expression profiling revealed similar chaotic expression patterns in DP and IDL leaves, which are labelled as I and II in Fig. 3. Group I exhibited different up/down-regulation in DPs and IDLs compared with pre-treatment. Group II showed a significant decrease in down-regulation in DP and IDL leaves compared with pre-treatment. Group III showed a significant increase in up-regulation in DPs and IDLs compared with pre-treatment. Also, group IV exhibited a similar expression pattern between DP and IDL leaves compared with pre-treatment (Fig. 3).

### Validation of microarray-based miRNAs

**Fig. 4:**
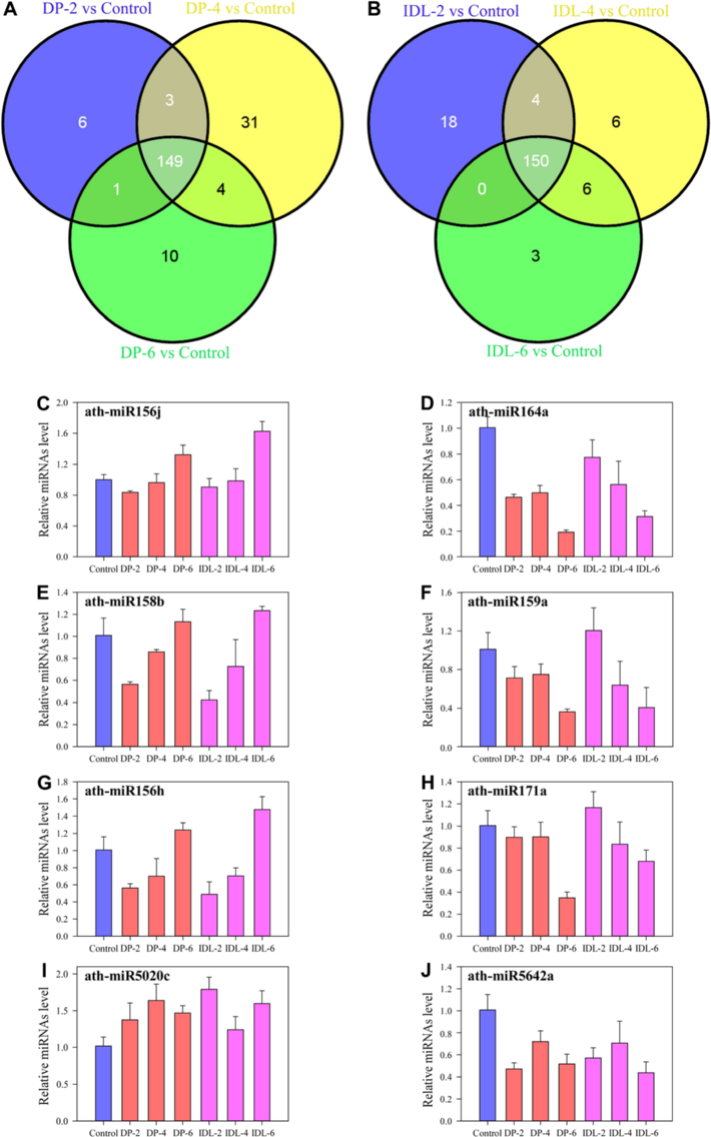
Venn diagram and real-time PCR analysis of differentially expressed miRNAs. **a** Venn diagram indicating DP and the control differentially expressed miRNAs in leaves after 2, 4 and 6d of dark-induced treatment. **b** Venn diagram indicating IDL and the control differentially expressed miRNAs in leaves after 2, 4 and 6d of individual dark-induced treatment. The number in the middle of the microarray and high-throughput sequencing circle represents miRNAs that had the same expression pattern in 2, 4 and 6d of DP (**a**) and IDL (**b**). The Venn diagram is the result with *P* < 0.01 in both experiments. **c**-**j** Quantitative analysis of eight miRNAs levels by stem-loop real-time RT-PCR in IDL and DP-induced leaves **c** miR156j, **d** miR164a, **e** miR158b, **f** miR159a, **g** miR156h, **h** miR171a, **i** miR5020c **j** miR5642a. UBQ6-1 was used as the internal control. Error bars indicate SD obtained from three biological repeats

Differential expression of the miRNAs in response to dark treatment was analysed for all miRNAs detected in the control, DPs (Fig. 4a) and IDLs (Fig. 4b). At 2, 4 and 6 d under dark-induced treatment, 159, 187 and 164 miRNAs were detected (signal intensity > 0), respectively. Among them, 149 miRNAs were expressed under DP, whereas 6, 31 and 10 were specifically expressed in 2, 4 and 6 d, respectively (Fig. 4a). However, this differed in IDLs (Fig. 4b). At 2, 4 and 6 d under IDL, 172, 166 and 159 miRNAs were detected, respectively. Among them, 150 miRNAs were expressed in all three samples, whereas 18, 6 and 3 were specifically expressed in 2, 4 and 6 d, respectively (Fig. 4b, Additional file 2: Table S3). Interestingly, 149 miRNAs expressed in all three samples in Fig. 4a are the same compared with Fig. 4b and 137 miRNAs had *P* < 0.01 (data not shown). Only ath-miR1886.1 is specifically expressed in 2, 4 and 6 d IDL (Additional file 2: Table S3). However, the expression of ath-miR1886.1 is very low and its signal intensity is under 50 in IDLs (data not shown).

We chose eight miRNAs with similar expression patterns in control, DP and IDL samples (shown as a red star in Fig. 3). The microarray analysis of the eight miRNAs in response to dark treatment was shown in Additional file 1: Figure S1. The expression levels of eight selected miRNAs were tested using quantitative real-time PCR (qRT-PCR), and confirmed the differential expression data obtained from microarray analysis on the whole (Fig. 4).We found the expression patterns of eight miRNAs were approximately consistent with the microarray data (Fig. 4, Additional file 1: Figure S3). The expressions of ath-miR164a, ath-miR159a, ath-miR171a and ath-miR5642a were down-regulated in both DP and IDL samples compared with the control (Fig. 4). Also, the expression of ath-miR5020c was up-regulated in both DPs and IDLs compared with the control. However, the expression of ath-miR156j, ath-miR158b, ath-miR156h and ath-miR5020c decreased before 4 d treatment and then increased in DP-6 and IDL-6d samples compared with the control (Fig. 4). Among them, over-expression of ath-miR164a has been previously reported to repress EIN3-induced early-senescence phenotypes [23]. ORE1/NAC2 was genetically identified as a positive regulator of leaf senescence, because knockout of ORE1/NAC2 extends plant longevity in *Arabidopsis* [25], and miR164 mediates the cleavage of a group of NAC family genes, of which ORE1/NAC2 is a positive regulator of aging-induced cell death and leaf senescence [25, 31].

### Analysis of miRNA target genes during dark-induced senescence characteristics

**Fig. 5:**
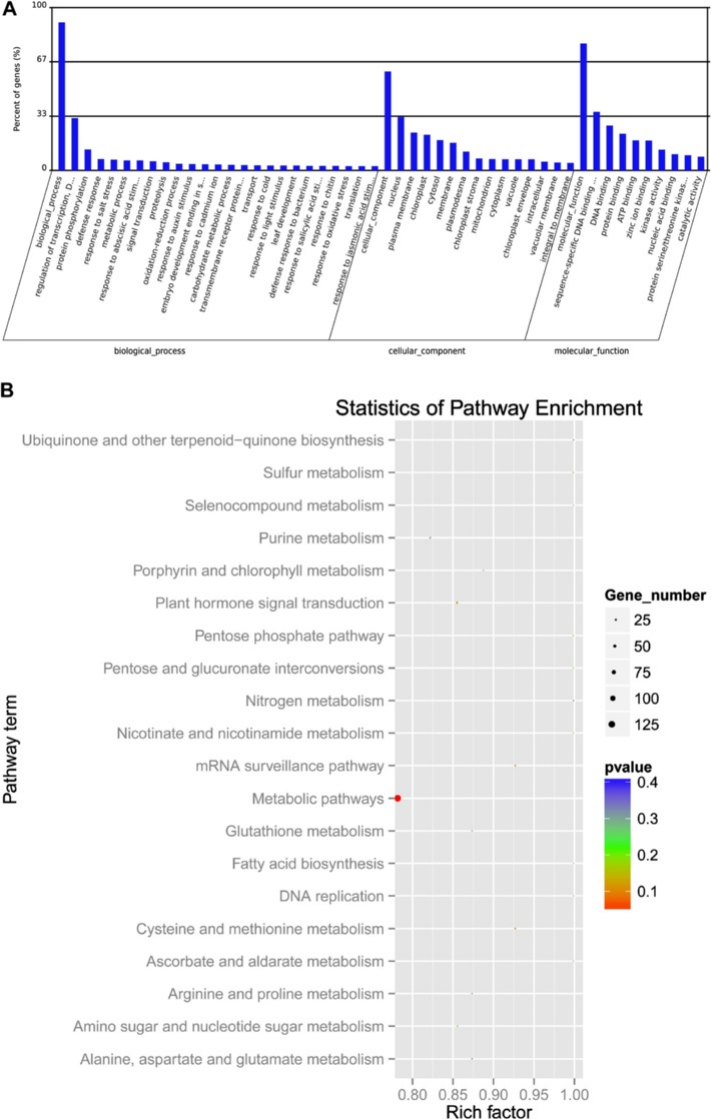
GO categories and KEGG pathway analysis for target genes of dark-induced senescence miRNAs. **a** GO categories for target genes of the miRNAs involved in biological processes, cellular components and molecular functions; **b** KEGG pathway analysis for target genes of miRNAs involved in processes such as metabolic pathways, plant hormone signal transduction, nitrogen metabolism and some biosynthesis pathways

After target gene prediction, we performed GO analysis on the predicted target genes of 137 differential miRNAs (*P* < 0.01) that changed in DP and IDL samples. We found the molecular functions of 1827 identified target genes to be involved in functions such as leaf development, gene silencing by miRNAs, response to auxin stimulus, response to salicylic acid stimulus, response to abscisic acid stimulus*, and so on* (Additional file 2: Table S4, data not shown). The results of GO analysis showed that the identified miRNAs and their targets were classified to 1584 GO terms including 867 biological processes, 174 cellular components and 543 molecular functions (Additional file 2: Table S4), and that the molecular functions of target genes were mainly focused on sequence-specific DNA binding, protein binding (Fig. 5a).

Pathway analysis based on the KEGG pathway database was also applied on predicted target genes of the differentially expressed miRNAs. After removing redundant terms, our findings pinpointed 98 annotated KEGG pathways (Additional file 2: Table S4) for the miRNAs and were enriched in 20 KEGG pathways (Fig. 5b). The KEGG enrichment analysis for target genes of miRNAs indicated that these genes regulated processes such as metabolic pathways, plant hormone signal transduction, nitrogen metabolism and some biosynthesis pathways (Fig. 5b).

### Differential expression analysis of targets during dark-induced senescence

**Fig. 6:**
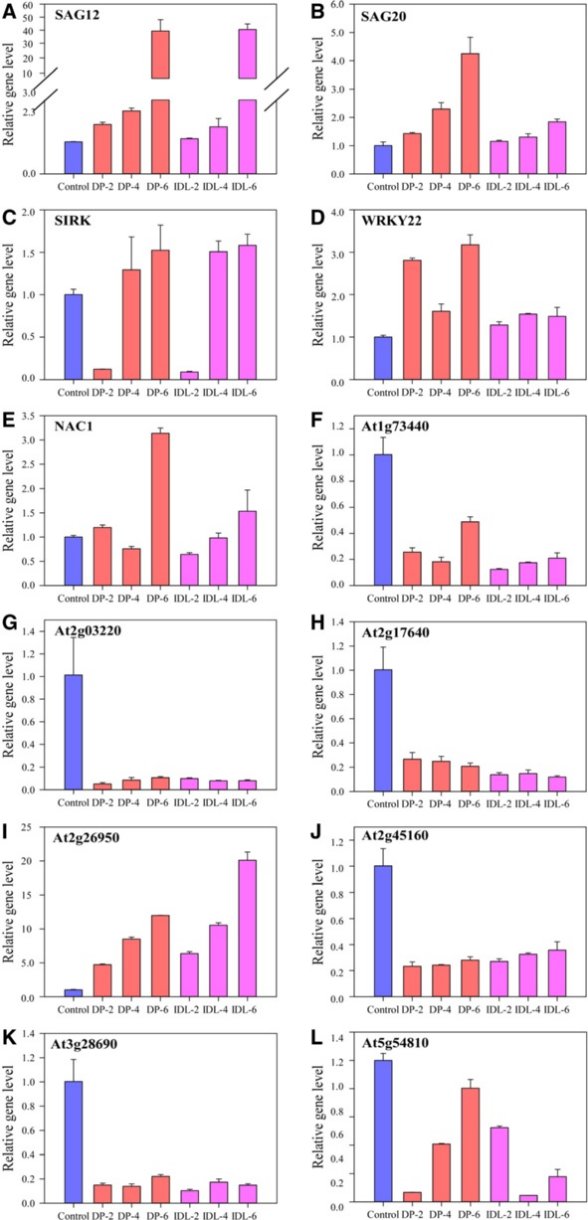
Real-time PCR analysis of differentially expressed target genes in DP and IDL *Arabidopsis* leaves. **a** SAG12, **b** SAG20, **c** SIRK, **d** WRKY22, **e** NAC1,**f** At1g73440, **g** At2g03220, **h** At2g17640, **i** At2g26950, **j** At2g45160; **k** At3g28690, **l** At5g54810. ACT2 gene was used as the internal control. Error bars indicate SD obtained from three biological repeats

In *Arabidopsis*, SAG12 expression is highly associated with age-regulated senescence and not induced by several stress conditions [8]. It is believed to be a reliable marker for natural leaf senescence [8, 32]. Also, SAG20, SIRK and WRKY22 are reportedly involved in leaf senescence [33, 34]. We found that the expression of these genes increased in all of the dark-induced samples (Fig. 6). Bioinformatics predictive analysis identified *NAC1*, *At1g73440* (calmodulin-related protein), *At2g03220* (galactoside 2-α-L-fucosyltransferase), *At2g17640* (serine acetyltransferase), *At2g26950* (MYB domain protein), *At2g45160* (protein lost meristems 1), *At28690* (putative protein kinase), *At5g54810* (tryptophan synthase beta chain) as target genes of miR164a, miR5020c, miR158b, miR156j, miR159a, miR171a, miR156h and miR5642a, respectively.

To confirm the causality of the miRNA expression patterns and its target gene, we studied the expression of these genes in the control, DP and IDL samples. *NAC1* and *At2g26950* expression levels were higher in DP/IDL than in the control which correlated with lower expression of miRNA164a and miR159a. miRNA164a expression level is higher in 6-DP than that in 6-IDL. Meantime, the other gene expression levels were lower in DP/IDL than in the control, correlating with higher expression of corresponding miRNAs (Fig. 6).

## Discussion

Leaf senescence is a natural age-dependent process. It can be affected through a complex regulatory network by internal and external signals, such as darkness, extreme temperature, drought and exposure to nutrient deficiency [7, 33]. Recently, by comparing transcriptome changes of 27 different treatments that are known to promote senescence, it was reported that the early pathways for the induction of senescence differ, but later converge into a shared senescence programme [35]. For many years it has been known that constant darkness can induce leaf senescence, and many studies of this phenomenon have been published [10, 13, 36, 37]. However, in almost all instances, the studies focused on either detached leaves [8, 31, 34, 38] or intact seedlings [13, 37, 39]. Little work has been done on IDLs in *Arabidopsis.* Furthermore, some reports have had somewhat contradictory conclusions, especially with regards to whether DP treatment can induce leaf senescence [13, 39, 40]. Weaver and Amasino concluded that darkness was the main factor causing senescence of individual leaves [40]. Recent studies found that more than 75 % of genes that are significantly up- or down-regulated in IDLs show the same response in natural and age-dependent senescence in *Arabidopsis.* This means that developmental senescence and dark-induced senescence programme with IDL treatment share many common pathways in *Arabidopsis* [10]. Based on these results, we used the fast, controlled and more synchronous induction of leaf senescence to mimic the developmental senescence by DPs or IDLs treatment.

Chl degradation is a visible symptom of leaf senescence, and the progress is usually detected by the amount of chlorophyll. Most reports agree that the transfer of whole plants to darkness induces chlorophyll loss in true leaves [8, 31, 41]. We found that dark-induced leaf senescence in *Arabidopsis* occurs more slowly in DPs relative to IDLs (Figs. 1 and 2). After dark treatment for 6d, both the Chl content of DP and IDL decreased to only ~25 % of the Control before treatment (Fig. 2). But, compared with the Control before treatment, WT was decrease ~10 % after 6 d growing under normal condition (Additional file 1: Figure S2). The senescence phenotype and Chl content differ to that described by others [14, 26, 37, 40], this maybe because seedlings were treated under the long-day photoperiod (16 h light/8 h dark) in our experiments, whereas in others seedlings were grown under a short-day photoperiod (8 h light/16 h dark) [14, 26]. Further, it maybe that different ecotypes result in a different phenotype [40].

A dark-induced senescence phenotype occurs more slowly than the molecular response in intact plants. With the completion of genome sequencing and the availability of several research tools, the molecular angle of dark-induced senescence in *Arabidopsis* has been thoroughly studied [26, 42, 43]. Previous studies have shown that epigenetic processes, which act as higher order regulatory switches in both developmental and stress-related induction of leaf senescence, play an important role in leaf senescence [44, 45]. Epigenetic regulated leaf senescence occurs mainly through changes in the chromatin structure, differential histone modifications, DNA methylation and small RNA binding/interaction [44–48]. Nevertheless, there are few reports of miRNA-controlled senescence [24–26, 49]. miR319 regulates leaf senescence by controlling TCP transcription factors. TCP transcription factor coordinates two sequential processes in leaf development (leaf growth) [48]. Kim *et al.* (2009) found that senescence was accelerated in the miR164 mutant [25]. They demonstrated that miR164 repressed ORE1 via cleavage of ORE1 mRNA. EIN3 and ORE1 can directly promote Chl degradation [50]. It was consistent with our data (Figs. 3 and 4). Also, ORE1 is a NAC transcription factor regulating downstream SAGs, such as SAG12 [35]. miR390 triggers the production of the trans-acting siRNA TAS3. TAS3 results in the mRNA degradation of ARF2 [49, 51]. ARF2 is a negative regulator of auxin responses, and auxin responses are involved in the timing of senescence [46, 52]. Moreover, miR-159a was found in leaf senescence of rice through genome-wide anlaysis of miRNAs [53].

Furthermore, the changes of miRNAs in dark-induced senescence in *Arabidopsis* remain unknown, especially experiments with both DP and IDL treatment. Weaver *et al.* (1998) found that in *Arabidopsis,* leaf senescence is not induced but is in fact inhibited when whole plants are placed in the darkness, whereas in contrast it is strongly accelerated when individual leaves are darkened while the rest of the plant remains in the light [8, 40]. This finding is consistent with our results in IDL but partly contradictory with the results in DP (Figs. 1, 3 and 4). In fact, in most cases , whole plants were treated with enduring darkness to induce senescence and found some SAG-related genes [8, 26]. Whether DP treatment can induce senescence is still unclear [10, 31, 40]. To resolve this, and to elucidate the molecular events in dark-induced senescence, we used microarray in DP and IDL in *Arabidopsis* to investigate the miRNAs and relative target genes.

In this study, 150 miRNAs were induced in all three DP samples and 149 miRNAs were expressed in all three IDL samples. Interestingly, 149 miRNAs were the same in both DP and IDL samples, indicating that the effects on leaf senescence of DP and IDL treatment are highly similar, and treatment of the entire plant in darkness also can induce senescence. Meantime, although the change tendency is similar, it has some difference between DP and IDL treatment, such as expression of miRNA159 in IDLs is more higher than that in DP samples, and so on (Fig. 6). Among the 149 senescence-related miRNAs, expression levels of 44 miRNAs were significantly altered in DPs and IDLs (Additional file 2: Table S3). Of these, six have been previously identified as being involved in senescence: miR319a, 319c, miR-159a, miR164a, miR164c and miR390a [25, 35, 46, 48, 49, 53]. Furthermore, we found that miR408 and miR396a are involved in leaf senescence (Additional file 2: Table S3), it was consistent with Thatcher’s deep sequence results [54]. We selected eight miRNAs from these 44 miRNAs to confirm the microarray data using qRT-PCR (Fig. 4) (miR164a, miR5020c, miR158b, miR156j, miR159a, miR171a, miR156h and miR5642a). miR164a is down-regulated in both DP and IDL leaves. It was shown that our data is consistent with age-associated leaf senescence in *Arabidopsis* [25]. The other 7 miRNAs, which were newly identified with qRT-PCR, were likely candidates involved in dark-induced senescence (Fig. 4).

Other studies have used microarray analysis to identify a large group of genes that show transcript level differences in response to dark treatment in *Arabidopsis* [13, 26]. Much research suggests that the leaf expression of a relatively large number of SAGs, such as SAG12, SAG20, SIRK, WRKY22 and NAC, are induced in response to darkness [8, 32, 33, 40, 43]. NAC is reportedly regulated by miR164 [3]. And miR159 was reported to determine leaf structure by targeting MYB [55]. We found these genes up-regulated in both DPs and IDLs, with the exception of SIRK which is down-regulated 2 d after treatment in DPs and IDLs (Fig. 6). This finding highlights that DP or IDL treatment can mimic age-associated senescence. Target predictions for the miRNAs covered in this study suggested the regulation of senescence processes, including leaf development, gene silencing by miRNA, and response to auxin stimulus (Fig. 5). We predicted the target gene of the remaining seven miRNAs selected in Fig. 4 and detected the expression of these genes (Fig. 6). Liu *et al.* (2011) have generated a leaf senescence database (LSD, http://www.eplantsenescence.org/) [56]. It comprises 1145 SAGs from 21 plant species. Li *et al*. (2012) also developed accurate database of genes potentially associated with leaf senescence in *Arabidopsis* [4]. Among the predicted target genes, we found that *At3g28690*, *At2g17640* and *At2g45160* are found in the LSD, which are target genes of miR-156h, miR-156j and miR-171a, respectively*. At1g73440*, *At2g03220*, *At5g54810* were not in the LSD, which were corresponding to the target genes of miR-5020c, miR-158b and miR-5642a, respectively. Target genes regulated by other 30 miRNAs in Additional file 2: Table S3, such as miR-158a, miR162a, miR166a, miR5021, miR171a, were involved in negative regulation of cell growth, growth rate, abscisic acid signaling pathway and light-harvesting complex II. These target genes were likely associated with leaf senescence. These miRNAs and target genes have not been published before. Our future studies will focus on the relationship between these miRNAs/target genes with leaf senescence.

## Conclusions

miRNAs responsive to dark-induced senescence were identified with the miRNA microarray. Furthermore, additional miRNA targets were predicted using bioinformatic approaches. A large number of miRNAs were induced or suppressed upon dark-induced senescence, showing miRNAs play an important role in leaf senescence. We can exclude some new miRNAs which regulate leaf senescence under IDL or DP treatment. Also we can identify likely target genes, which may have negative or positive effects on the dark-induced leaf senescence. This study further expands on the existing knowledge of the roles of miRNAs in dark-induced leaf senescence and provides a number of new miRNAs to explore in the future.

## Availability of supporting data

The data set supporting the results of this article is available in the NCBI GEO (Gene expression omnibus, http://www.ncbi.nlm.nih.gov/geo/) repository under the accession number of GSE74376.

## Additional files

Additional file 1: Figure S1. Analysis of differentially expressed miRNAs according the microarray platform in IDL and DP-induced leaves. (A) miR156j; (B) miR164a; (C) miR158b; (D) miR159a; (E) miR156h; (F) miR171a; (G) miR5020c; (H) miR5642a. Error bars indicate SD obtained from three biological repeats. (DOC 224 kb) Additional file 2:
**In this additional table, it includes 4 sub-tables. **All the annotations of the tables are followings, also in the additional table. **Table S1.** Quantitative RT-PCR Primer sequences. **Table S2.** miRNAs change after darkened treatment (p < 0.01, Signal insensity > 500). **Table S3.** Common miRNAs change during darkness treatment (Signal insensity > 500). **Table S4.** Dark-induced senescence miRNAs, miRNA targets, GO terms and KEGG pathways in DP/IDL *Arabidopsis* leaves. (DOC 224 kb)
