# Supplementary material for: Identification of miRNAs associated with dark-induced senescence in Arabidopsis
Source: BMC Plant Biol. 2015 Nov 4;15:266. doi: 10.1186/s12870-015-0656-5 (PMC4632659; doi:10.1186/s12870-015-0656-5)
Supplement: Additional file 1: Figure S1. — Analysis of differentially expressed miRNAs according the microarray platform in IDL and DP-induced leaves. (A) miR156j; (B) miR164a; (C) miR158b; (D) miR159a; (E) miR156h; (F) miR171a; (G) miR5020c; (H) miR5642a. Error bars indicate SD obtained from three biological repeats. (DOC 224 kb) [file 12870_2015_656_MOESM1_ESM.doc]

**Additional file 1:**

**
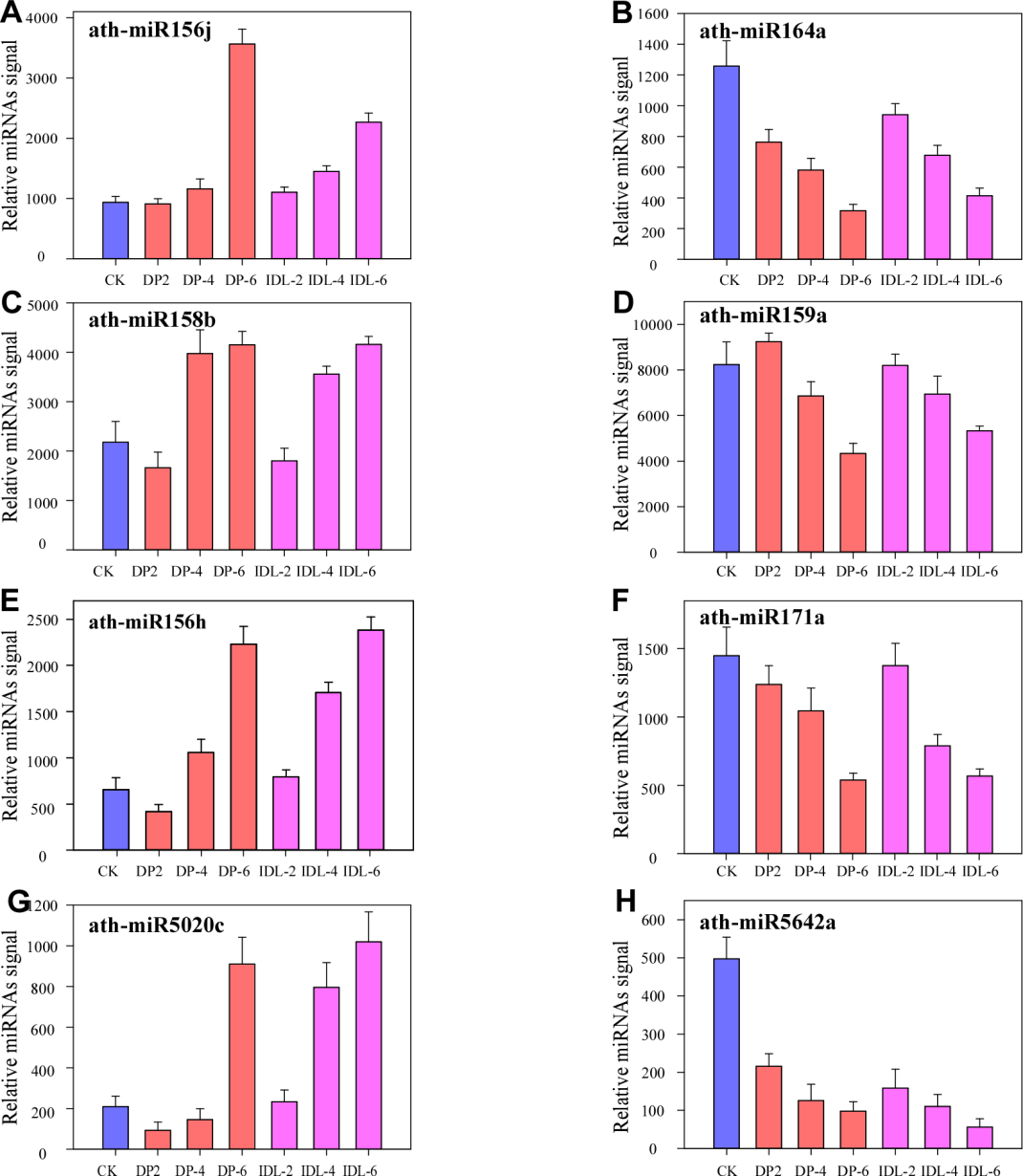
**

**Figure S1.** Analysis of differentically expressed miRNAs according the microarray platform in IDL and DP-induced leaves. (A) miR156j; (B) miR164a; (C) miR158b; (D) miR159a; (E) miR156h; (F) miR171a; (G) miR5020c; (H) miR5642a. Error bars indicate SD obtained from threebiological repeats.
