# Supplementary material for: Identification of miRNAs associated with dark-induced senescence in Arabidopsis
Source: BMC Plant Biol. 2015 Nov 4;15:266. doi: 10.1186/s12870-015-0656-5 (PMC4632659; doi:10.1186/s12870-015-0656-5)
Supplement: Additional file 2: — In this additional table, it includes 4 sub-tables. All the annotations of the tables are followings, also in the additional table. Table S1. Quantitative RT-PCR Primer sequences. Table S2. miRNAs change after darkened treatment (p < 0.01, Signal insensity > 500). Table S3. Common miRNAs change during darkness treatment (Signal insensity > 500). Table S4. Dark-induced senescence miRNAs, miRNA targets, GO terms and KEGG pathways in DP/IDL Arabidopsis leaves. (DOC 224 kb) [file 12870_2015_656_MOESM2_ESM.doc]

**Additonal file 2:**

**Table S1.** Quantitative RT-PCR Primer sequences.

| Locus Name | Alias | Primers | Sequece (5’->3’) |
| --- | --- | --- | --- |
| X52527.1 | UBQ6-1 | Forward  Reverse | CCGATAAAATTGGAACGATACAG  ATTTCTCGATTTATGCGTGTCA |
| NC_016089 | ath-miR156j | Forward  Reverse | GGCGGCGTGACAGAAGAGA  CAGTGCAGGGTCCGAGGTAT |
| AT1G55591 | ath-miR158b | Forward  Reverse | GGTCCCCAAATGTAGACAAAGC  CAGTGCAGGGTCCGAGGTAT |
| AT1G73687 | ath-miR159a | Forward  Reverse | GGCGGTTTGGATTGAAGG  CAGTGCAGGGTCCGAGGTAT |
| AT2G47585 | ath-miR164a | Forward  Reverse | CGGCGTGGAGAAGCAGG  CAGTGCAGGGTCCGAGGTAT |
| AT3G51375 | ath-miR171a | Forward  Reverse | CAGCAGTGATTGAGCCGC  CAGTGCAGGGTCCGAGGTA |
|  | ath-miR5642a | Forward  Reverse | GGAGGATCTCGCGCTTGTA  CAGTGCAGGGTCCGAGGTAT |
| AT5G55835 | ath-miR156h | Forward  Reverse | GGCGGTGACAGAAGAAAGAGAG  CAGTGCAGGGTCCGAGGTAT |
|  | ath-miR5020c | Forward  Reverse | CGGTGGCATGGAAGAAGGT  CAGTGCAGGGTCCGAGGTAT |
| AT4G23810 | WRKY53 | Forward  Reverse | GATCACAAGAACACCACCATTAGCC  AAAGTTGTGTCAATCTCGACCGTTG |
| AT3G10985 | SAG20 | Forward  Reverse | TCGGTAACGTTGTTGCTGGA  ACCAAACTCTTTCAAATCGCCA |
| AT3G18780 | ACT2 | Forward  Reverse | TGTGCCAATCTACGAGGGTTT  TTTCCCGCTCTGCTGTTGT |
| AT5G45890 | SAG12 | Forward  Reverse | TCCAATTCTATTCGTCTGGTGTGT  CCACTTTCTCCCCATTTTGTTC |
| AT1G17290 | 156h | Forward  Reverse | TTCTGCTCCTTCCGATTCTT  TCTCCACGGACAGCATACTC |
| AT1G27360 | 156h | Forward  Reverse | CCATATCGTGAATGGACTGC  ACATGATCAAATGCTCCCAA |
| AF486619  (At2g19190) | SIRK | Forward  Reverse | AGCAGCTCAATTAAGTAAATGGCG  CCCGCAATCTATACTTATGAAACCA |
| AT4G01250 | WRKY22 | Forward  Reverse | CGACAAAGTAATGCCGTCTCC  CGTTTCTGGTTCTGTGGCTTT |
| AT1G56010 | AtNAC1 | Forward  Reverse | TGGGAGGGAAGGATTGGTATT  GCCCCGTCGCGTATTTT |
| AT3G28690 | 156h | Forward  Reverse | CGATCAAAGGTGCTCAGAAA  GGCTTCCACAACTTCACTCA |
| AT2G17640 | 156j | Forward  Reverse | GCTGCAGGTTCACTTGTGTT  GCTAGAGACGGGTCTTGCTC |
| AT2G03220 | 158b | Forward  Reverse | CTTTCACCAACACCCATCTG  GGCGTCGAGAACTCTAGCTT |
| AT2G26950 | 159a | Forward  Reverse | AGAACTTGGAGCTCCCTTCA  GGCCTATCAGAGGAGACTGG |
| AT4G15530 | 159a | Forward  Reverse | TGGTTCGAACTGAGACAAGC  AACAACCAGCAATGCAACAT |
| AT4G37770 | 159a | Forward  Reverse | AGAATGCTATGGCGGATTT  GTCTCGTTAGCCGGAGTAGC |
| AT2G45160 | 171a | Forward  Reverse | CGATTTCGACGTTGGATATG  AGAAGGTGGAGGAGCAAAGA |
| AT1G73440 | 5020c | Forward  Reverse | AGTTTCCCTTGGTGAGGATG  GTCATCTGCGTCTTGCTCAT |
| AT1G03940 | 5020c | Forward  Reverse | ATCAATGTGGATTGCAGGAA  TGATACGATACCAGGAGCCA |
| AT1G12663 | 5020c | Forward  Reverse | TTCAACAACCTTCTCCACCA  GCTGATACTCTTTCCACATTCG |
| AT1G64200 | 5642a | Forward  Reverse | TTCCTCCTGCTCCTTCAGAT  AGTTTATTGCGGAAGGCAAC |
| AT5G54810 | 5642a | Forward  Reverse | GATTGGACAGTGGCAAACAC  CGACTCCAGGGTAGTCCAAT |

**Table S2.** miRNAs change after darkened treatment (signal>500, P<0.01).

|  | **Probe ID Signal value (mean±SD)** | | | | | | |  |
| --- | --- | --- | --- | --- | --- | --- | --- | --- |
| **Reporter**  **Name** | **CK(mean±SD)** | **DP2(mean±SD)** | **DP4(mean±SD)** | **DP6(mean±SD)** | **IDL2(mean±SD)** | **IDL4(mean±SD)** | **IDL6(mean±SD)** | **P-value(P< 0.01)** |
| ath-miR167a | 9,590±301 | 10,081±206 | 9,348±1125 | 6,900±337 | 10,968±698 | 9,444±187 | 7,789±215 | 0.00E+00 |
| ath-miR167d | 9,139±430 | 9,977±209 | 9,403±762 | 6,695±307 | 10,690±653 | 9,162±170 | 7,481±428 | 0.00E+00 |
| ath-miR159b | 8,789±504 | 9,337±291 | 6,915±559 | 4,493±288 | 8,270±506 | 7,147±122 | 5,383±179 | 0.00E+00 |
| ath-miR173-5p | 7,167±321 | 7,051±165 | 5,418±1269 | 4,340±210 | 6,351±253 | 4,539±89 | 4,018±85 | 0.00E+00 |
| ath-miR5021 | 1,612±201 | 1,462±192 | 2,070±545 | 4,391±519 | 1,921±173 | 3,750±178 | 3,769±148 | 0.00E+00 |
| ath-miR396a | 1,598±291 | 1,000±221 | 1,621±489 | 2,724±257 | 1,767±165 | 4,184±160 | 4,097±289 | 0.00E+00 |
| ath-miR156j | 874±234 | 913±42 | 1,161±156 | 3,565±229 | 1,106±60 | 1,361±321 | 2,270±108 | 0.00E+00 |
| ath-miR822 | 2,338±298 | 3,562±66 | 2,045±231 | 1,375±94 | 3,096±110 | 1,816±61 | 1,226±65 | 0.00E+00 |
| ath-miR396b | 1,023±151 | 522±144 | 1,060±329 | 1,952±254 | 1,051±107 | 3,066±201 | 3,144±295 | 0.00E+00 |
| ath-miR156h | 656±102 | 414±64 | 1,056±136 | 2,231±174 | 793±60 | 1,707±61 | 2,384±103 | 0.00E+00 |
| ath-miR156i | 575±174 | 514±37 | 647±280 | 2,144±199 | 752±39 | 1,228±62 | 1,643±106 | 0.00E+00 |
| ath-miR5658 | 334±101 | 327±43 | 603±77 | 1,401±233 | 457±52 | 841±48 | 986±58 | 0.00E+00 |
| ath-miR3440b-3p | 108±27 | 111±45 | 430±143 | 1,006±357 | 224±53 | 933±103 | 838±38 | 0.00E+00 |
| ath-miR164c | 1,164±216 | 791±46 | 591±60 | 315±21 | 919±92 | 671±24 | 408±12 | 0.00E+00 |
| ath-miR164a | 1,163±355 | 764±62 | 581±63 | 317±24 | 943±31 | 677±19 | 414±22 | 0.00E+00 |
| ath-miR5020c | 210±39 | 93±25 | 145±48 | 910±117 | 233±50 | 795±101 | 1,020±130 | 0.00E+00 |
| ath-miR5655 | 22±5 | 43±13 | 61±14 | 307±62 | 61±13 | 185±25 | 453±107 | 0.00E+00 |
| ath-miR5642a | 497±67 | 216±13 | 126±34 | 97±14 | 158±42 | 110±10 | 56±10 | 0.00E+00 |
| ath-miR398b | 247±39 | 240±19 | 168±42 | 102±12 | 294±58 | 531±18 | 304±13 | 0.00E+00 |
| ath-miR2936 | 115±22 | 77±10 | 173±32 | 324±34 | 95±9 | 338±32 | 477±34 | 0.00E+00 |
| ath-miR398a | 243±29 | 225±21 | 147±35 | 97±13 | 292±8 | 507±19 | 300±12 | 0.00E+00 |
| ath-miR171a | 1,449±169 | 1,158±299 | 917±332 | 539±29 | 1,376±147 | 789±52 | 568±20 | 9.99E-16 |
| ath-miR159a | 8,241±804 | 9,244±275 | 6,397±1727 | 4,333±323 | 8,196±467 | 6,941±415 | 5,329±147 | 4.33E-15 |
| ath-miR165a | 3,833±188 | 3,309±143 | 3,240±459 | 3,172±914 | 2,115±168 | 3,609±500 | 4,169±329 | 3.24E-14 |
| ath-miR408 | 368±88 | 387±27 | 360±147 | 530±49 | 731±50 | 828±22 | 822±56 | 5.38E-13 |
| ath-miR163 | 1,821±638 | 1,956±105 | 1,467±156 | 1,242±118 | 1,879±428 | 871±117 | 824±23 | 1.29E-12 |
| ath-miR172c | 1,716±141 | 1,988±108 | 2,223±482 | 2,443±73 | 1,695±153 | 1,496±64 | 2,079±97 | 2.53E-12 |
| ath-miR400 | 533±101 | 324±61 | 540±141 | 700±68 | 560±47 | 601±40 | 642±57 | 7.10E-12 |
| ath-miR395b | 291±91 | 52±11 | 48±11 | 167±33 | 450±1320 | 239±28 | 215±32 | 2.00E-11 |
| ath-miR160a | 545±163 | 766±31 | 526±76 | 754±52 | 844±37 | 542±25 | 558±19 | 6.82E-11 |
| ath-miR162a | 550±118 | 450±37 | 437±46 | 401±31 | 593±80 | 487±32 | 441±47 | 3.90E-10 |
| ath-miR157d | 1,655±258 | 2,270±62 | 2,194±419 | 2,110±149 | 2,317±374 | 1,559±87 | 1,825±63 | 1.49E-09 |
| ath-miR172a | 1,830±281 | 2,307±124 | 2,333±555 | 2,540±98 | 1,861±197 | 1,547±83 | 2,173±112 | 2.97E-09 |
| ath-miR390a | 899±187 | 1,087±43 | 907±275 | 983±30 | 657±59 | 517±21 | 659±25 | 2.57E-08 |
| ath-miR158b | 2,179±377 | 1,660±311 | 3,652±1213 | 4,152±260 | 1,803±246 | 3,559±77 | 4,161±68 | 3.76E-08 |
| ath-miR319a | 1,539±482 | 1,202±199 | 1,603±427 | 1,536±109 | 1,626±86 | 2,282±100 | 1,976±230 | 4.77E-06 |
| ath-miR156g | 1,113±195 | 1,147±37 | 1,359±175 | 1,288±221 | 1,199±123 | 983±53 | 1,241±73 | 5.04E-06 |
| ath-miR161.2 | 525±118 | 531±41 | 623±75 | 668±29 | 720±53 | 537±118 | 529±46 | 5.08E-06 |
| ath-miR167c | 7,218±696 | 8,379±192 | 7,104±2471 | 5,927±318 | 9,342±618 | 7,806±109 | 6,665±272 | 3.16E-05 |
| ath-miR171b | 441±108 | 471±19 | 376±76 | 336±20 | 452±23 | 498±44 | 448±31 | 5.61E-05 |
| ath-miR157a | 1,661±510 | 2,310±87 | 2,244±301 | 2,173±97 | 2,493±201 | 1,639±50 | 1,875±66 | 1.29E-04 |
| ath-miR166a | 4,975±1826 | 5,032±181 | 5,273±624 | 5,308±912 | 4,107±883 | 5,925±180 | 6,671±372 | 1.09E-03 |
| ath-miR319c | 3,777±1409 | 4,299±302 | 3,577±915 | 2,616±218 | 4,300±160 | 3,898±188 | 3,084±378 | 1.66E-03 |
| ath-miR403 | 3,302±940 | 2,952±185 | 2,306±1305 | 3,988±160 | 3,154±368 | 2,786±142 | 3,561±265 | 2.50E-03 |

**Table S3.** Common miRNAs change during darkness treatment (Signal>500).

| **Number** | **Common elements**  **in "IDL-2 vs CK", "IDL-4 vs CK" and "IDL-6 vs CK"** | **Common elements**  **in "DP-2 vs CK", "DP-4 vs CK" and "DP-6 vs CK"** |
| --- | --- | --- |
| 1 | ath-miR156a | ath-miR156a |
| 2 | ath-miR156g | ath-miR156g |
| 3 | ath-miR156h | ath-miR156h |
| 4 | ath-miR156i | ath-miR156i |
| 5 | ath-miR156j | ath-miR156j |
| 6 | ath-miR157a | ath-miR157a |
| 7 | ath-miR157d | ath-miR157d |
| 8 | ath-miR158a | ath-miR158a |
| 9 | ath-miR158b | ath-miR158b |
| 10 | ath-miR159a | ath-miR159a |
| 11 | ath-miR159b | ath-miR159b |
| 12 | ath-miR159c | ath-miR159c |
| 13 | ath-miR160a | ath-miR160a |
| 14 | ath-miR161.1 | ath-miR161.1 |
| 15 | ath-miR161.2 | ath-miR161.2 |
| 16 | ath-miR162a | ath-miR162a |
| 17 | ath-miR163 | ath-miR163 |
| 18 | ath-miR164a | ath-miR164a |
| 19 | ath-miR164c | ath-miR164c |
| 20 | ath-miR165a | ath-miR165a |
| 21 | ath-miR166a | ath-miR166a |
| 22 | ath-miR167a | ath-miR167a |
| 23 | ath-miR167c | ath-miR167c |
| 24 | ath-miR167d | ath-miR167d |
| 25 | ath-miR168a | ath-miR168a |
| 26 | ath-miR169a | ath-miR169a |
| 27 | ath-miR169b | ath-miR169b |
| 28 | ath-miR169d | ath-miR169d |
| 29 | ath-miR169h | ath-miR169h |
| 30 | ath-miR170 | ath-miR170 |
| 31 | ath-miR171a | ath-miR171a |
| 32 | ath-miR171b | ath-miR171b |
| 33 | ath-miR172a | ath-miR172a |
| 34 | ath-miR172b-5p | ath-miR172b-5p |
| 35 | ath-miR172c | ath-miR172c |
| 36 | ath-miR172e | ath-miR172e |
| 37 | ath-miR173-3p | ath-miR173-3p |
| 38 | ath-miR173-5p | ath-miR173-5p |
| 39 | **ath-miR1886.1** | ath-miR1886.3 |
| 40 | ath-miR1886.3 | ath-miR1888a |
| 41 | ath-miR1888a | ath-miR2111a-5p |
| 42 | ath-miR2111a-5p | ath-miR2112-5p |
| 43 | ath-miR2112-5p | ath-miR2934-5p |
| 44 | ath-miR2934-5p | ath-miR2936 |
| 45 | ath-miR2936 | ath-miR2937 |
| 46 | ath-miR2937 | ath-miR319a |
| 47 | ath-miR319a | ath-miR319c |
| 48 | ath-miR319c | ath-miR3434-3p |
| 49 | ath-miR3434-3p | ath-miR3440b-3p |
| 50 | ath-miR3440b-3p | ath-miR390a |
| 51 | ath-miR390a | ath-miR391 |
| 52 | ath-miR391 | ath-miR3933 |
| 53 | ath-miR3933 | ath-miR393a |
| 54 | ath-miR393a | ath-miR394a |
| 55 | ath-miR394a | ath-miR395a |
| 56 | ath-miR395a | ath-miR395b |
| 57 | ath-miR395b | ath-miR396a |
| 58 | ath-miR396a | ath-miR396b |
| 59 | ath-miR396b | ath-miR397b |
| 60 | ath-miR397b | ath-miR398a |
| 61 | ath-miR398a | ath-miR398b |
| 62 | ath-miR398b | ath-miR399e |
| 63 | ath-miR399e | ath-miR399f |
| 64 | ath-miR399f | ath-miR400 |
| 65 | ath-miR400 | ath-miR403 |
| 66 | ath-miR403 | ath-miR404 |
| 67 | ath-miR404 | ath-miR405a |
| 68 | ath-miR405a | ath-miR406 |
| 69 | ath-miR406 | ath-miR408 |
| 70 | ath-miR408 | ath-miR414 |
| 71 | ath-miR414 | ath-miR415 |
| 72 | ath-miR415 | ath-miR416 |
| 73 | ath-miR416 | ath-miR418 |
| 74 | ath-miR418 | ath-miR4221 |
| 75 | ath-miR4221 | ath-miR4227 |
| 76 | ath-miR4227 | ath-miR4239 |
| 77 | ath-miR4239 | ath-miR4243 |
| 78 | ath-miR4243 | ath-miR4245 |
| 79 | ath-miR4245 | ath-miR447a.2-3p |
| 80 | ath-miR447a.2-3p | ath-miR447a-3p |
| 81 | ath-miR447a-3p | ath-miR472 |
| 82 | ath-miR472 | ath-miR5015 |
| 83 | ath-miR5015 | ath-miR5017 |
| 84 | ath-miR5017 | ath-miR5018 |
| 85 | ath-miR5018 | ath-miR5020b |
| 86 | ath-miR5020b | ath-miR5020c |
| 87 | ath-miR5020c | ath-miR5021 |
| 88 | ath-miR5021 | ath-miR5023 |
| 89 | ath-miR5023 | ath-miR5025 |
| 90 | ath-miR5025 | ath-miR5026 |
| 91 | ath-miR5026 | ath-miR5028 |
| 92 | ath-miR5028 | ath-miR5029 |
| 93 | ath-miR5029 | ath-miR5634 |
| 94 | ath-miR5634 | ath-miR5635a |
| 95 | ath-miR5635a | ath-miR5636 |
| 96 | ath-miR5636 | ath-miR5642a |
| 97 | ath-miR5642a | ath-miR5643a |
| 98 | ath-miR5643a | ath-miR5645a |
| 99 | ath-miR5645a | ath-miR5647 |
| 100 | ath-miR5647 | ath-miR5648-5p |
| 101 | ath-miR5648-5p | ath-miR5649a |
| 102 | ath-miR5649a | ath-miR5650 |
| 103 | ath-miR5650 | ath-miR5651 |
| 104 | ath-miR5651 | ath-miR5653 |
| 105 | ath-miR5653 | ath-miR5654-3p |
| 106 | ath-miR5654-3p | ath-miR5655 |
| 107 | ath-miR5655 | ath-miR5657 |
| 108 | ath-miR5657 | ath-miR5658 |
| 109 | ath-miR5658 | ath-miR5659 |
| 110 | ath-miR5659 | ath-miR5660 |
| 111 | ath-miR5660 | ath-miR5664 |
| 112 | ath-miR5664 | ath-miR5665 |
| 113 | ath-miR5665 | ath-miR5996 |
| 114 | ath-miR5996 | ath-miR5998a |
| 115 | ath-miR5998a | ath-miR5999 |
| 116 | ath-miR5999 | ath-miR771 |
| 117 | ath-miR771 | ath-miR773a |
| 118 | ath-miR773a | ath-miR774a |
| 119 | ath-miR774a | ath-miR774b-3p |
| 120 | ath-miR774b-3p | ath-miR775 |
| 121 | ath-miR775 | ath-miR776 |
| 122 | ath-miR776 | ath-miR778 |
| 123 | ath-miR778 | ath-miR780.2 |
| 124 | ath-miR780.2 | ath-miR822 |
| 125 | ath-miR822 | ath-miR823 |
| 126 | ath-miR823 | ath-miR824 |
| 127 | ath-miR824 | ath-miR825 |
| 128 | ath-miR825 | ath-miR827 |
| 129 | ath-miR827 | ath-miR828 |
| 130 | ath-miR828 | ath-miR829.1 |
| 131 | ath-miR829.1 | ath-miR829.2 |
| 132 | ath-miR829.2 | ath-miR830-3p |
| 133 | ath-miR830-3p | ath-miR833a-3p |
| 134 | ath-miR833a-3p | ath-miR834 |
| 135 | ath-miR834 | ath-miR835-3p |
| 136 | ath-miR835-3p | ath-miR841a |
| 137 | ath-miR841a | ath-miR841b-3p |
| 138 | ath-miR841b-3p | ath-miR841b-5p |
| 139 | ath-miR841b-5p | ath-miR843 |
| 140 | ath-miR843 | ath-miR845a |
| 141 | ath-miR845a | ath-miR847 |
| 142 | ath-miR847 | ath-miR848 |
| 143 | ath-miR848 | ath-miR851-3p |
| 144 | ath-miR851-3p | ath-miR854a |
| 145 | ath-miR854a | ath-miR857 |
| 146 | ath-miR857 | ath-miR862-3p |
| 147 | ath-miR862-3p | ath-miR863-3p |
| 148 | ath-miR863-3p | ath-miR863-5p |
| 149 | ath-miR863-5p | ath-miR867 |
| 150 | ath-miR867 |  |

**Table S4.** Dark-induced senescence miRNAs, miRNA targets, GO terms and KEGG pathways in DP/IDL *Arabidopsis* leaves.

| Total miRNAs (p<0.01) | Target genes (miRNA number) | GO terms (1417) | | | Target genes in annotated KEGG Pathways (miRNA number) | Annotated KEGG Pathways |
| --- | --- | --- | --- | --- | --- | --- |
|  |  | Cellular component | Biological process | Molecular function |  |  |
| 137 | 1827 (123) | 174 | 867 | 584 | 278(60) | 98 |
